# Myocardial arterial spin labeling

**DOI:** 10.1186/s12968-016-0235-4

**Published:** 2016-04-12

**Authors:** Frank Kober, Terrence Jao, Thomas Troalen, Krishna S. Nayak

**Affiliations:** Aix-Marseille Université, CNRS CRMBM UMR 7339, Centre de Résonance Magnétique Biologique et Médicale, Marseille, France; Department of Biomedical Engineering, University of Southern California, Los Angeles, California USA; Ming Hsieh Department of Electrical Engineering, University of Southern California, Los Angeles, California USA

**Keywords:** Myocardial perfusion, Arterial spin labeling, Cardiovascular magnetic resonance, Ischemic heart disease

## Abstract

Arterial spin labeling (ASL) is a cardiovascular magnetic resonance (CMR) technique for mapping regional myocardial blood flow. It does not require any contrast agents, is compatible with stress testing, and can be performed repeatedly or even continuously. ASL-CMR has been performed with great success in small-animals, but sensitivity to date has been poor in large animals and humans and remains an active area of research. This review paper summarizes the development of ASL-CMR techniques, current state-of-the-art imaging methods, the latest findings from pre-clinical and clinical studies, and future directions. We also explain how successful developments in brain ASL and small-animal ASL-CMR have helped to inform developments in large animal and human ASL-CMR.

## Background

**Fig. 1:**
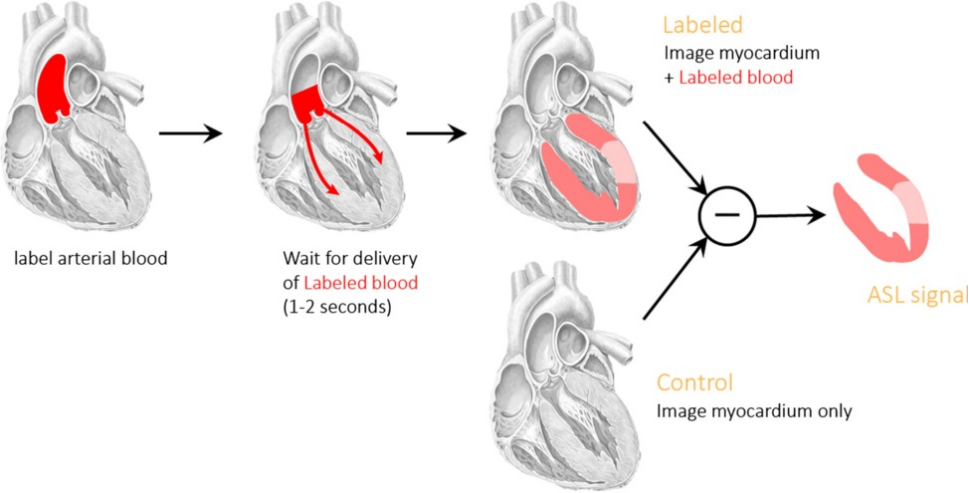
ASL is a subtraction technique. Images are acquired (top) with and (bottom) without a preceding RF pulse that labels inflowing blood. The difference between these two images can be made directly proportional to myocardial blood flow in units of mL-blood/g-tissue/min. (Figure courtesy of Eric C. Wong, MD, PhD, University of California, San Diego)

Arterial spin labeling is a cardiovascular magnetic resonance (CMR) technique for quantifying tissue blood flow, non-invasively and without contrast agents [1, 2]. Radiofrequency pulses are used to modify the longitudinal magnetization of arterial blood, creating an endogenous label (or tracer) that decays with the time-constant equal to the T_1_ relaxation time, about 1.5 seconds for blood at 3 Tesla. After a delay to allow labeled blood to flow into the target tissue, images are acquired that reflect inflow of labeled blood as well as static tissue whose magnetization exchanges with that of the inflowing blood. A second set of images is acquired in the absence of a preceding labeling pulse. The difference between these two image sets reflects the amount of labeled blood that has been delivered to the imaging region, and with appropriate labeling, imaging, and perfusion model, can be made directly proportional to tissue blood flow (in mL-blood per g-tissue per minute). This process is illustrated in Fig. 1.

For myocardial perfusion applications, ASL is an attractive alternative to first-pass CMR and traditional nuclear medicine for two reasons. First, it does not require intravenous contrast agents or ionizing radionuclides, and therefore can be performed repeatedly or even continuously with no incremental risk to the patient. Second, it provides measurements that are directly proportional to myocardial blood flow as opposed to a relative measurement that requires designating an area of normal myocardium for comparison [3, 4]. Therefore, ASL could be more sensitive for detection of disease that reduces perfusion globally such as balanced ischemia, microvascular disease and multi-vessel disease.

### Review

First-pass gadolinium enhanced perfusion CMR has a long history in both clinical research and diagnostic routine, mainly for the evaluation of ischemic heart disease [5]. Its superiority for diagnosing CAD compared with SPECT was shown in several large multi-center trials including CE-MARC and MR-IMPACT [6–8]. First-pass CMR owes its success mainly to its high spatial resolution compared with nuclear imaging techniques. Also, the contrast change produced by the contrast agent bolus during a perfusion measurement is high (of the order of 60 % with saturation-recovery SSFP techniques in normal myocardium [9]), the delineation of territories with perfusion defects can be done without sophisticated post-processing tools. The standard approach for clinical interpretation is visual delineation of hypo-perfused territories.

Quantitative or at least a semi-quantitative analysis of perfusion is desirable particularly in non-ischemic cardiomyopathies affecting microvasculature, which cause only subtle and non-focal defects. While first-pass CMR does allow quantification of blood flow, the mathematical modeling of contrast dynamics is relatively complex and hasn’t yet found consensus in the community. Absolute quantification with first-pass CMR requires a measurement of the Arterial Input Function (AIF), which has to be obtained without saturation of the contrast/concentration curve. This implies the use of either dual-contrast pulse sequences [10] or dual-bolus protocols [11, 12]. First-pass CMR is therefore an excellent and widely recognized method for identifying and delineating altered tissue in ischemic heart disease thanks to very good detection sensitivity. Robust absolute MBF quantification approaches are, however, still subject of ongoing research.

One important limitation of first-pass CMR stems from the use of Gadolinium-based contrast agents (GBCA), which have known toxicity in patients with renal failure. In these patients, excessive use can lead to organ fibrosis and failure. GBCA’s are contraindicated in patients with end-stage renal disease (ESRD), which is a small but significant part of the CMR patient population, due to their need for frequent cardiovascular assessment. ESRD prevalence is on the rise, with an annual growth rate of 3–4 % in the US [13], and ~7 % worldwide [14]. These patients would benefit from a new safer alternative, as they require ischemic heart disease testing every 12 months to a) determine preoperative cardiovascular risk prior to kidney transplant surgery and b) because they have >10 times higher cardiovascular mortality than the general population [15, 16]. These patients cannot receive GBCA because of poor kidney function and experience an unreasonably large radiation dose from frequent stress testing using SPECT or PET [17–19]. Today, many centers use a less sensitive dobutamine stress echo test to screen for cardiovascular disease in ESRD patients.

ASL-CMR is a totally non-invasive alternative that uses magnetically labeled blood as an endogenous tracer. It would therefore offer accessibility of perfusion CMR to patients with contraindications to GBCA. ASL-CMR is therefore increasingly relevant in patients with Chronic Kidney Disease (CKD), the elderly in general, and the growing number of patients who simply refuse diagnostic tests involving contrast or radiation.

The contrast differences created by magnetic labeling of blood are inherently limited, and ASL therefore features much lower sensitivity than 1^st^ pass techniques. The signal in ASL is indeed directly proportional to myocardial blood flow. The myocardial tissue magnetization difference created by magnetic blood labeling depends on the labeling type and is lower than 4 % in humans (as an example: 1.5 % [20]). However, for a number of reasons, absolute quantification with ASL is at least theoretically more reliable and more accurate: 1) The created bolus of labeled magnetization is short and its spatial profile is almost perfectly rectangular, 2) the timing and shape of this bolus do not vary between experiments, 3) the signal to concentration relation is perfectly linear, since only water is used as tracer, and 4) the measurement is freely repeatable without bias across consecutive measurements.

The repeatability of ASL-CMR is particularly interesting for rest/stress comparisons, since measurements could be carried out in a continuous way to monitor tissue blood flow changes during stress. Furthermore, the possibility to do absolute quantification would make ASL a good candidate for longitudinal therapeutic monitoring and disease follow-up studies.

### From the brain to the heart

ASL methods for measuring cerebral blood flow are robust and widely used in the research setting, for example, to assess stroke [21, 22], and functional brain activation [23–25]. A recent consensus document has provided recommended protocols that are available on commercial platforms and has received broad support from the developer and user community [26]. The success and maturity of ASL in the brain has provided significant motivation for ASL-CMR and has provided a significant toolbox of techniques that may be applied in ASL-CMR.

There are several important physiological and practical factors that necessitate the use of different ASL labeling and imaging methods for measuring myocardial blood flow (MBF) and cerebral blood flow (CBF), respectively. These are summarized in Table 1, and discussed briefly here. Tissue blood flow is higher in the myocardium compared to the brain, roughly 2x higher at rest and 5–7x higher during vasodilation, resulting in a stronger ASL signal. Water exchange kinetics of labeled spins to tissue water are assumed to be instantaneous in the heart while the blood-brain barrier (BBB) decreases the exchange rate of water in the brain. The upstream pathway that blood travels to perfuse the myocardium is complicated compared to the brain. This influences scan timing, and the selection of labeling geometries as blood that perfuses the myocardium may have been in the imaging volume a few seconds prior. The heart is also moving rapidly and blood flow is highly pulsatile. This requires labeling and imaging methods that are synchronized to the cardiac cycle. Imaging is typically performed during quiescent periods, end-systole and mid-diastole. The intrinsic SNR efficiency of modern MRI systems with the latest in receiver coil technology is 3.5x lower in the heart compared to the brain (for normal weight subjects). This is due largely to the need for large coil arrays, with greater distance between coil and tissue, and larger noise volume. This SNR penalty is greater for overweight and obese subjects. Finally, the optimal imaging methods must provide highest possible tissue SNR efficiency, which is influenced by T1 and T2, which are different for myocardium compared to gray/white matter. These differences are all critical for the design of ASL-CMR methods, and for understanding how they subtly and significantly differ from brain ASL methods.

**Table 1:** Factors that impact the use of ASL in heart and in the brain, for measuring myocardial blood flow (MBF) and cerebral blood flow (CBF), respectively

	Heart (MBF)	Brain (CBF)
Tissue Blood Flow	0.5 – 1.5 mL/g/min (rest) 3.0 – 5.0 mL/g/min (stress)	0.35 – 0.55 mL/g/min [100]
Water Kinetics	Instantaneous exchange	Decreased water exchange due to blood brain barrier (BBB)
Blood pathway	Many directions; Blood passes through left atrium, left ventricle, aortic root, coronary arteries and then myocardium.	Unidirectional; Internal carotids and vertebral arteries deliver blood to the Circle of Willis, then to branch vessels, then to the brain. Blood flowing primarily inferior to superior.
Motion	Significant cardiac motion; Quiescent periods are: End systole: ~80 ms long Mid diastole: 0-150 ms long depending on heart rate. Respiratory motion must be handled either by limiting the acquisition time to a breath-hold or by correcting for motion	Insignificant motion compared to the spatial resolution typically used (~2 mm).
Intrinsic SNR efficiency	1/3 of Brain (normal weight subjects); due to larger distance from receiver coil elements and larger noise volume	
Imaging Concerns	Imaging must be performed during quiescent cardiac phases.	
Labeling Concerns	Direction of Flow Pulsatile Flow Cardiac Motion	Maximum efficiency (pseudo-continuous labeling recommended [26])
Optimal Readouts	For maximum myocardial signal: Snapshot balanced SSFP Single-shot EPI	For maximum gray/white matter signal: Single-shot Spiral Single-shot EPI

Many of the fundamental developments in ASL were first applied and validated in the brain. Of particular relevance, two signal models have been developed for extracting tissue blood flow from ASL signals. The “dual T1” approach by Detre et al. and the general kinetic model by Buxton et al. have both been used in ASL-CMR [27–29]. Methods for pulsed labeling, including methods that balance magnetization transfer effects, were first developed in the brain and have been applied in ASL-CMR [29, 30]. And several other methods have not yet been successfully used in ASL-CMR, but show promise. These include: background suppression (to reduce physiological noise), velocity-selective labeling (to provide measurements independent of transit delay), pseudo-continuous labeling (to maximize labeling efficiency), and simultaneous multi-slice imaging (to broaden spatial coverage without increasing scan time).

### From mouse to man

Interestingly, perfusion MRI including perfusion CMR has gone in two completely different directions for clinical and pre-clinical application. The reasons for this become clear when comparing the major morphologic, physiologic and MRI-technical factors that differ between humans and rodents, summarized in Table 2. Beyond the obvious differences in size, the rodent heart is characterized by dramatically higher heart rate, cardiac index and blood turnover in the body. Another interesting difference is given by the flow velocities in large vessels, which are indeed comparable between humans and rodents despite the difference in body size. Myocardial blood flow is roughly five times higher in rodents than in humans. These given facts have consequences for the design and the feasibility of CMR techniques in rodents in general and for perfusion CMR techniques in particular.

**Table 2:** Comparison of typical major cardiovascular features between humans and rodents. The values for rats and mice are average values typical for animals under isoflurane anesthesia. The differences in blood recirculation time and capillary blood flow have strong impact on the choice of methods for measuring perfusion

	Human	Rat	Mouse
Body weight	80 kg	0.3 kg	0.03 kg
Total blood volume	5 L	18 mL	1.8 mL
End diastolic volume (EDV)	180 mL	300 μL	50 μL
Myocardial Mass	150 g	0.6 g	0.1 g
Heart Rate	80 bpm	300 bpm	600 bpm
Cardiac Output	5 L/min	0.15 L/min	0.020 L/min
Time of recirculation	60 s	7 s	5 s
Aortic ejection velocity [101–103]	100 cm/s	200 cm/s	300 cm/s
Breathing rate	12 bpm	70 bpm	110 bpm
Myocardial perfusion (MBF)	1 mL/g/min	5 mL/g/min	7 mL/g/min

For first-pass CMR, the fact that blood circulation times in rodents are very short requires short bolus injection durations with high demand on the power injector performance for generating a reproducible shape. It also requires very rapid imaging techniques for sampling the AIF with sufficient time resolution. Fast imaging is also required due to the high heart rates. 1^st^ pass imaging in the mouse heart became indeed only feasible by the use of accelerated acquisition techniques [7, 8]. Bolus injection volumes in the mouse are of the order of 100 μL injected at a flow rate of 2 mL/min [31]. A second difficulty is given by the high capillary blood flow itself, which produces rather sharp signal increases during first pass. This makes the deconvolution techniques used for perfusion quantification less reliable. Nevertheless, an important advantage of first-pass techniques is their speed. Reported acquisition times are of the order of 60 s in the mouse heart [32].

For ASL-CMR in the rodent heart, the short blood traveling times and high capillary blood flow are beneficial, since the signal used for quantification is directly proportional to MBF. It does not suffer from limitations related to bolus sharpness and reproducibility, since the generated magnetic labeling zone has a perfectly defined shape and negligible duration. Since the labeling can be unconditionally repeated, k-space acquisition can be segmented, and the spatial resolution is therefore not limited by acquisition speed requirements. However, even with the high MBF in rodents, the produced contrast (approximately 20 %) is still much lower than that produced by GBCA. Both the segmentation used for producing high-resolution images and the comparatively small signal therefore lead to long acquisition times compared with first-pass CMR; 4–20 min have been reported [33–36]. In humans, ASL-CMR is more challenging given the lower blood flow values and heart rates. Also, since respiration gating in humans is not as regular and reliable as in animals, the scans are in general performed under breath-hold, which limits the available acquisition time.

### ASL-CMR in rodents

The feasibility of ASL was first demonstrated in the rodent brain [27, 37], and application to the perfused excised rat heart was also in the focus of the very first ASL studies [38]. Since then, a significant amount of work has been carried out on methods for ASL-CMR in rodents in vivo.

**Fig. 2:**
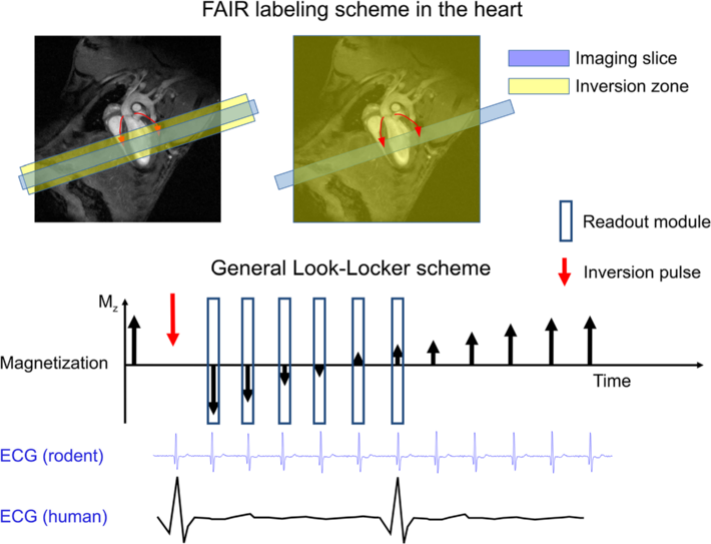
The FAIR labeling scheme (upper part) consists in two inversion recovery measurements. In the first measurement the labeling zone is only around the imaging slice, and in the second measurement the inversion is global. FAIR is the most commonly used ASL technique in the heart. The timing of the inversion and that of the readout modules should be ECG-gated and occur in the same cardiac phase. The Look-Locker inversion-recovery readout (lower part) has been used mainly (although not exclusively) in the rodent heart, where the rapid heart rate permits dense sampling of the magnetization recovery curve. Due to the lower heart rate in humans, only one readout at a specific inversion time is generally performed

Most studies have used the flow-sensitive alternating inversion recovery (FAIR) scheme [39] consisting of a pair of global and slice-selective inversion recovery experiments Fig. 2. Combined with a Look-Locker readout scheme (LLFAIR), absolute MBF quantification was shown feasible by comparison of T1 relaxation times measured with both experiments. The first successful report of MBF mapping in rats by Belle et al. [33] employed a LLFAIR snapshot-FLASH approach. These authors used a two-compartment quantification model that was initially setup with the goal to correct for errors induced by perfusion on myocardial blood volume measurements [40, 41], and which resulted in a slightly modified relation between perfusion and slice-selective and global inversion tissue T1 values from the initial Detre relation [27]. The study was done in animals under artificial respiration that was arrested during image acquisition to avoid artifacts.

**Fig. 3:**
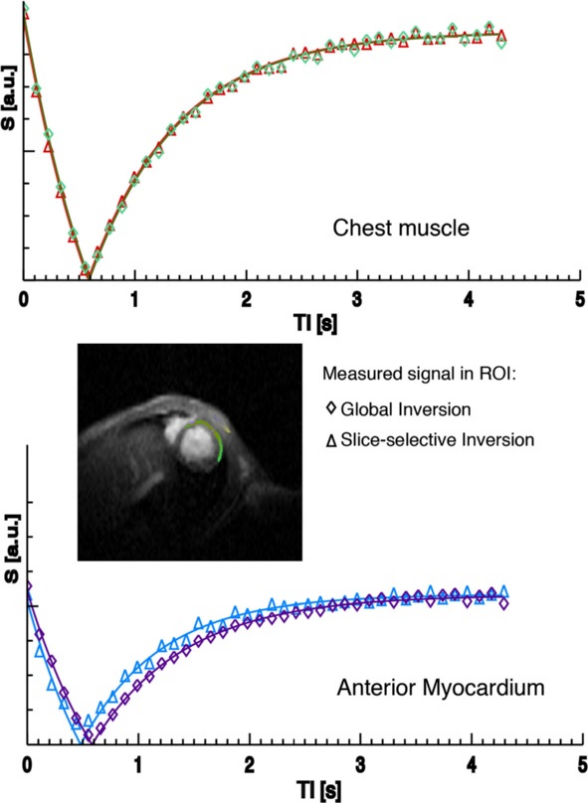
Look-Locker FAIR recovery signal behavior in myocardial and chest muscle regions in a mouse at 4.7 T (magnitude data, symbols represent the average of pixel signals in each ROI after global or slice-selective inversion, continuous line is an exponential fit for T1_gl_ and T1_sl_). The signal difference due to perfusion is nearly absent in the very weakly perfused chest muscle, whereas myocardium shows a clear difference in relaxation. Data: CRMBM Marseille

More recent efforts have improved the robustness of MBF quantification using a respiration- and ECG-gated LLFAIRGE single-gradient echo technique [34, 42], which allowed for higher spatial resolutions, freely breathing animals and reduced blurring. These improvements were particularly useful in the mouse heart, which features very high heart rates making snapshot acquisitions prone to blurring [43]. As a drawback, this led to significantly increased measurement times of about 25 min. An example showing the magnetization recovery for two different regions of interest in a mouse is shown in Fig. 3. It can be seen that the Look-Locker approach is advantageous in a small animal context, since – owing to the high heart rate - magnetization recovery can be sampled with good temporal resolution. One more recent improvement of LLFAIRGE incorporated better handling of respiratory motion and heart rate variations using fuzzy C-means clustering but did not shorten the acquisition duration [44].

Campbell-Washburn et al. have suggested several improvements of LLFAIRGE. A segmented k-space acquisition was done using a data logger in addition for recording individual repetition times [35]. The same group also showed the feasibility of multi-slice ASL in mice [45] using a segmented LLFAIR method, in which they also concentrated on ways to take into account the influence of partial saturation of the LV chamber blood by the image acquisition itself. Finally, a detailed analysis of potential error sources in LLFAIR measurements has recently been published by Kampf et al. [46].

Abeykoon et al. [47] proposed a single-inversion-time FAIR-ASL method instead of the T1-based methods used beforehand. Similar approaches had been used earlier, but in the human heart [48]. In the mouse heart, the authors found lower standard deviations within myocardial regions of interest on the perfusion maps than with the well-established LLFAIRGE technique, while absolute quantification of MBF led to similar values.

The FAIR technique is a pulsed ASL (PASL) technique, which has limited acquisition efficiency compared with the continuous ASL (CASL) techniques well known from brain studies [37]. To benefit from the sensitivity advantages of CASL in CMR applications, a steady-pulsed ASL (spASL) scheme using a cine readout incorporating one labeling pulse per cardiac cycle (cine-ASL) was suggested [36, 49]. This steady-pulsed ASL method was shown to allow shorter acquisition times (8 min) than the LLFAIR technique while preserving signal to noise ratio, spatial resolution and robustness with respect to cardiac motion, since no k-space segmentation was used. Unlike other continuous ASL techniques, a drawback of cine-ASL is that a separate T1 measurement is necessary for absolute quantification, but it is an efficient technique in particular when repeated measurements are required during a single exam, such as in rest/stress comparisons. This technique was also shown to be able to detect perfusion variations across the cardiac cycle [50].

### Validation studies

All small animal ASL-CMR validation studies to-date were performed in rats. Waller et al. [51] provided validation of the snapshot-FLASH FAIR Look Locker technique against microspheres in pentobarbital-anesthetized rats at rest and under adenosine stress and found good correlation between both techniques. Jacquier et al. [52] validated the LLFAIRGE technique in rats under isoflurane anesthesia at rest and under stress, although in two different groups of animals undergoing MRI and microsphere surgery. The latter study reported much larger perfusion reserve and MBF, which was attributed to the differences in the anesthetics used. However, quantitative MBF values were similar between microspheres and ASL-CMR.

### First-pass CMR in rodents

Despite the technical challenges, small-animal first-pass bolus tracking techniques were recently shown feasible thanks to efforts made in accelerating image acquisition. Makowski et al. [53] introduced a *k-t* SENSE technique for rodent myocardial blood flow (MBF) measurements on a clinical 3 T scanner. First-pass CMR was performed based on a saturation recovery spoiled gradient echo method with 10-fold *k*-space and time domain undersampling (*k-t* PCA, [54]), and the technique was validated in healthy mice by comparison with fluorescent microspheres [55] at rest and during dipyridamole-induced vasodilator stress. In parallel, Coolen et al. [32] proposed another approach using a four-element detection coil and GRAPPA *k*-space acceleration allowing for semi-quantitative assessment of the myocardial perfusion status. Naresh and coworkers recently used BLOSM-accelerated 1^st^ pass CMR to study myocardial blood flow in a high-sucrose diet diabetic mouse model [56]. The same group has provided a systematic comparison of this approach with a FAIR Look-Locker technique in terms of image quality, quantification and reproducibility [57].

### Performance

Among the published methods, it is difficult to identify a method of choice in general terms. Table 3 provides an overview over the major published methods along with their advantages and drawbacks. In a recent study by Naresh et al. [57], a BLOSM-accelerated 1^st^ pass technique was compared with a FAIR-spiral ASL technique pointing out that 1^st^ pass CMR had advantages in low blood flow situations whereas ASL gave better perfusion mapping quality and better reproducibility when high blood flow was high.

**Table 3:** Rodent ASL-CMR methods used in different studies along with their advantages and drawbacks

Method	Post-processing	Advantages	Drawbacks	Author
LLFAIR-snap	T_1_^sl^ / T_1_^gl^ maps pixel fit sat. correction	short acq. time good accuracy T1 map included	low res. low SNR	Belle [33], Waller [51, 58], Hiller [104]
Seg. LLFAIR LLFAIRGE	T_1_^sl^ / T_1_^gl^ maps pixel fit sat. correction	high res. good accuracy T1 map included	long acq. time	Kober [34, 42] Campbell-Washburn [35, 45], Streif [43], Vandsburger [44, 60], Caudron [61]
FAIR-1TI	difference image	short acq. time	unknown T1	Abeykoon [47]
cine-ASL	difference image	short acq. time high res. cine aspect	unknown T1 unknown labeling efficiency	Troalen [36, 50]

### Interesting recent findings

There are a number of examples using ASL in rodent models of cardiovascular disease. The topic of infarction and the role of microcirculation in infarction has been extensively studied particularly in rat models [51, 58, 59]. Among the more recent ones, Vandsburger et al. [60] have studied MBF along with many other parameters including regional function in a nNOS^-/-^ knockout mouse model. They found normal perfusion reserve under β-adrenergic stimulation despite a severely altered functional response. In another study, both relaxation times and perfusion were analyzed in spontaneously hypertensive rats at various stages of the disease [61]. Along with correlations of T_1_ and T_2_ with fibrotic content obtained by histology, the authors showed that MBF decreased in hypertensive rats without any correlation between perfusion and capillary density of the myocardium. Namely, anti-hypertensive therapy was shown to produce a significant improvement of myocardial perfusion in this model.

Several studies focusing on the infarcted heart made use of LLFAIR ASL. For example, the impact of endothelial cell transplantation in the infarcted myocardium was investigated by Zhang et al. [62]. MBF was quantified with and without transplantation of human endothelial cells into the infarcted heart, and a significant perfusion increase was observed in the treated hearts. This perfusion improvement was thus accompanied by an increase of microvasculature density in the infarcted regions. ASL CMR was also able to show improved myocardial perfusion after myocardial infarction in mice with PTP1B deficiency. The improved perfusion was likely due to an enhancement of angiogenesis in this genetically modified mouse model [63].

**Fig. 4:**
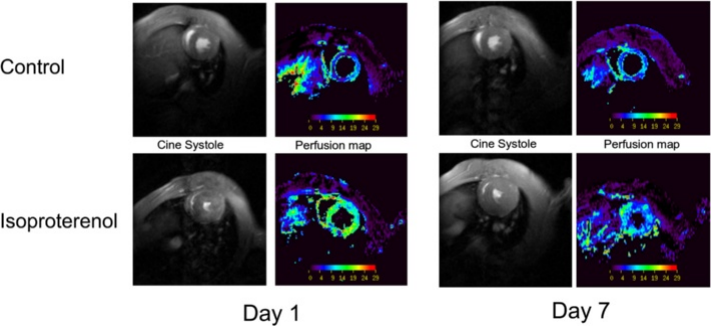
Myocardial perfusion and cardiac function under continuous isoproterenol administration in rats. Both perfusion and function are strongly increased by this inotropic agent shortly after infusion start (day 1). The strong contractility is sustained over seven days while perfusion diminishes to lower values leading to morphological alterations at longer term (adapted from Desrois et al. [65])

Absolute MBF quantification using LLFAIR was shown to be a unique and sensitive index to evaluate therapy in rodent heart disease models. Another therapy based on soluble epoxide hydrolase inhibition, which increases cardiovascular protective acids, has been shown to exert beneficial effects in a chronic heart failure model [64]. Increased perfusion in infarcted myocardium was found to be a short-term process preceding the long-term effects of LV remodeling. In a study exploring overstimulation of the rat heart by chronic infusion of isoproterenol, perfusion was shown to accompany the strong workload increase only temporarily, such that a chronic mismatch between workload and oxygen supply occurred, which resulted in reduced cardiac performance after one week of isoproterenol administration [65] (Fig. 4). More recently, the quantification capabilities of ASL have been used in a longitudinal study using multiple CMR modalities in a high-fat high-sucrose mouse model of type 2 diabetes [66] to monitor microvascular changes induced by the modified diet.

### ASL-CMR in large animals and humans

A different set of ASL pulse sequences are used in large animals and humans because of their vastly lower heart rates and perfusion when compared to small animals; these sequences are summarized in Table 4.

**Table 4:** Large Animal and Human ASL-CMR methods used in different studies. To date, with exception of the spASL technique, all studies used the FAIR labeling approach combined with different readout strategies

Author	Year	Post-labeling delay	Labeling method	Imaging method	Quantification
Capron [75]	2014	HR dependent	spASL	bSSFP	spASL
Wang [20]	2010	200 - 1700 ms	FAIR	bSSFP	Signal Difference
Zun [48]	2009	1 RR	FAIR	bSSFP	Signal Difference
Northrup [76]	2007	(1-12)RR + 91-177 ms	FAIR	GRE	Dual T1
Wacker [69]	2003	100 -1400 ms	FAIR-sat	FLASH	Dual T1
Poncelet [67]	1999	1 RR	FAIR	EPI	Signal Difference

The first published ASL studies in the human heart were independently carried out by Poncelet et al. [67] and Wacker et al. [68]. Poncelet et al. used a FAIR-EPI technique on a clinical 3 T system with a single-loop receive coil. Two cardiac-gated EPI readouts were performed after slice-selective and global inversion pulses, and the individual inversion times were recorded and used in the quantification model. The authors used the original quantification model by Detre et al. [27] approximating relaxation times of blood and tissue as equal. Physiological noise was assessed and considered as a major challenge. The sequence was also used in pigs under rest and stress conditions, and the ASL results were compared with microspheres showing a moderate correlation. As a result, the technique was considered to give reliable measurements, but only under pharmacologic stress conditions, in which blood flow is high. In parallel, Wacker and coworkers published ASL-CMR data from humans using a saturation-recovery snapshot FLASH technique at 1.5 T using a 4-channel array. They performed 9 cardiac-gated readouts per slice-selective or global saturation and used a slightly modified approach for quantification via fitted global and slice-selective T1 values [33]. In a second study, Wacker et al. [69] used a similar technique at 2 Tesla in CAD patients and were able to show reduced perfusion reserve in affected myocardial territories.

Zun et al. [48] later provided an experimental measurement metric for physiological noise based on signal variation across repetitions. They employed a single-TI FAIR bSSFP technique at 3 T. Wang et al. [20] employed a similar but navigator-gated approach with FAIR labeling under free breathing conditions. They used a non-rigid motion correction algorithm inspired from 1^st^ pass CMR perfusion sequences to correct for residual motion [70]. The sequence was performed at multiple labeling delay times to allow for simultaneous quantification of myocardial blood flow and arterial transit time (ATT). They reported resting perfusion values that compared favorably with perfusion values reported in the literature from PET [71, 72] and first-pass perfusion MRI [73]. Miyazaki et al. adapted a time-spatial inversion pulse (time-SLIP) for non-contrast angiography to ASL perfusion at multiple labeling delay times followed by 3D bSSFP imaging [74]. In the labeled condition, a non-selective inversion was followed by a slice-selective inversion centered on the aortic root while in the control condition, a single non-selective inversion pulse was used. They observed a transit time of 200–400 ms from the aortic root to the imaging volume.

A new free-breathing labeling scheme called steady pulsed ASL (spASL) was developed by Capron et al. [75] for the heart that brought the advantage of high signal to noise found in continuous ASL to a pulsed ASL technique. Labeling and snapshot bSSFP imaging were performed every cardiac cycle during end-systole and mid-diastole respectively to drive the perfusion signal to a steady state. Two cardiac gated image series were acquired under labeled and control conditions. The labeling slab was placed at the coronary root while the control slab was placed symmetrically about the imaging slice in a manner similar to EPISTAR. The authors found that spASL had higher SNR compared to FAIR while maintaining similar perfusion quantification. The spASL method will, however, require further improvements regarding motion management of labeling and acquisition to become fully efficient.

### Myocardial Blood Flow (MBF) quantification

Quantification of FAIR ASL falls into two main categories that either use a signal intensity difference method or a dual T1 approach. Two variations of the difference method have emerged. In its original implementation, both the labeling pulse and the image acquisition were gated to the same cardiac phase in consecutive heartbeats. While this ensured that labeling and image acquisition excited the same volume of heart, it also introduced a changing labeling time (TI) from heart rate variation that prevents direct subtraction of global and slice-selective image pairs. Instead, myocardial data along with two additional images at a short and long TI were fit to a three parameter inversion recovery model to generate a global and a slice-selective inversion recovery curve [67]. The signal difference was subsequently extrapolated at the average labeling time.

**Fig. 5:**
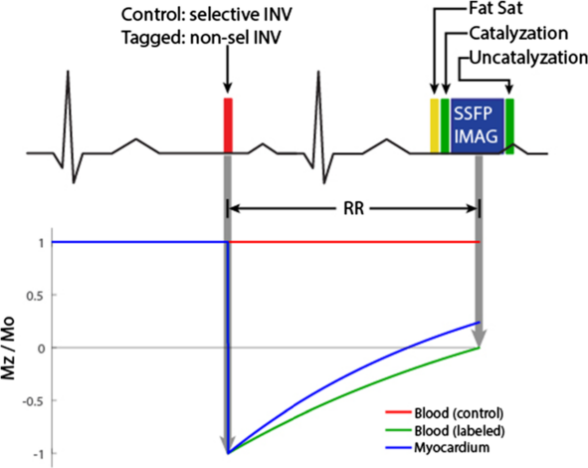
ECG-gated FAIR Pulse Sequence Diagram with single inversion time readout mainly (but not exclusively) used in the human heart. Labeling and imaging are placed in the same cardiac phase to ensure the same volume of myocardium is excited. Imaging is preceded by a fat saturation and an initial preparation consisting of a catalyzation ramp to reduce transient oscillations

Zun et al. [48] and Wang et al. [20] used a simplified version of the difference method that kept the TI constant between pairs of control and slice-selective images [48]. The pulse sequence they used is shown in Fig. 5. While this allows for direct subtraction of image pairs, it is more susceptible to quantification errors when heart rate variations are ≥ 4 bpm between image pairs (unpublished data). Wang et al. [20] found that non-rigid motion correction was able to mitigate quantification error and improve reliability.

The dual T1 approach to perfusion quantification was first formulated by Detre et al. [27] and experimentally verified by Bauer et al. [41] in an isolated perfused cardioplegic rat heart. Wacker et al. [69] used a saturation recovery FLASH sequence at nine different saturation delays for T1 quantification. They reported overestimated perfusion values, potentially related to the fact that the labeling saturation was done in varying cardiac phases. Northrup et al. [76] used a Look-Locker inversion recovery sequence to ensure that labeling and imaging occurred in the same cardiac phase. However, the authors noted that in a study by Zhou et. al [77], dual T1 quantification can either underestimate or overestimate FAIR perfusion in the presence of transit delay while the difference approach can only underestimate perfusion.

**Fig. 6:**
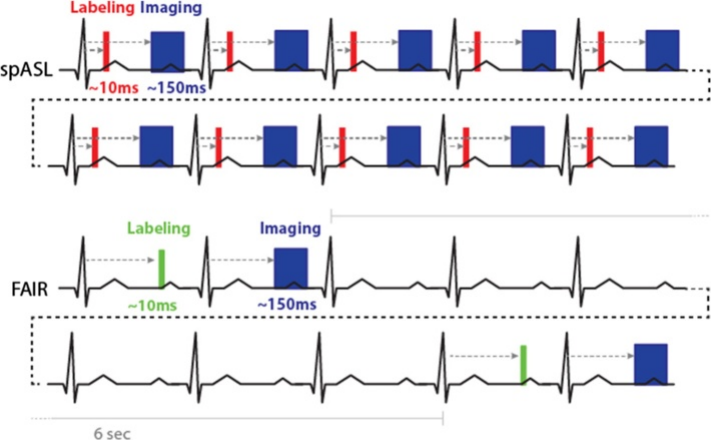
Acquisition timing diagram for spASL and FAIR ASL-CMR. In spASL, labeling and imaging occur every heartbeat during end-systole and mid-diastole, respectively. In FAIR, labeling and imaging occur in consecutive heartbeats at mid-diastole. An additional 6 sec wait is required to allow for T1 recovery of the label before the next acquisition. spASL has approximately 5x higher scan efficiency than FAIR, but is also more sensitive to gating errors and heart rate variability

Quantification of perfusion with spASL [49] takes into account the influence of multiple labeling pulses that are applied to blood as it travels from the left ventricular blood pool to the capillary bed of the myocardium. The steady state signal evolution changes depending on the image acquisition scheme and is incorporated into the quantification equation. By acquiring a large number of images, images that are mistriggered or corrupted by respiratory motion can be rejected. Figure 6 compares the pulse sequence of spASL and FAIR.

### Registration

Image registration has proven to be an important step in the analysis of ASL-CMR images, primarily because it is a subtraction technique, so small spatial errors can result in errors in perfusion quantification and/or increased physiological noise. This is very important during free-breathing acquisitions [20], but also has benefits during breath-held acquisitions [78]. There are a variety of established techniques for registering CMR images that can be directly applied to ASL-CMR images [79–82].

### Performance/interesting findings

In large animals, MBF measurements based on FAIR Look-Locker gradient-echo ASL-CMR have correlated well with microsphere histology [83]. Zhang et al. measured perfusion at rest and under dipyridamole induce stress in two healthy dogs and four dogs with 70 % occlusion to the left circumflex artery. Correlation between MRI and microsphere measured perfusion were 0.9 and 0.63 for myocardium supplied by normal and stenotic vessels respectively.

**Fig. 7:**
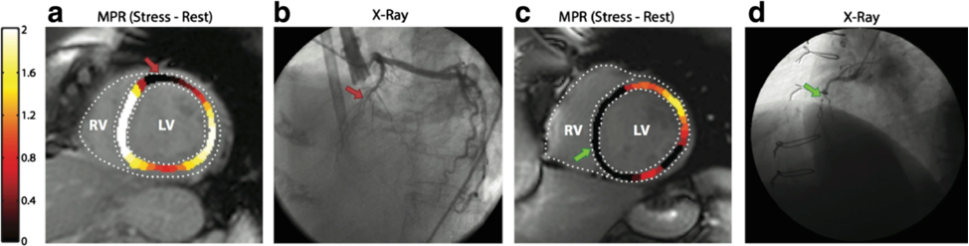
ASL-CMR in patients with single-vessel CAD. Patient with total LAD occlusion shows reduced perfusion reserve by (**a**) ASL consistent with (**b**) angiography (red arrows). Patient with total RCA occlusion shows reduced perfusion reserve by (**c**) ASL consistent with (**d**) angiography (green arrows). The myocardial perfusion reserve (MPR) color scale represents 0.0 – 2.0 ml/g/min. (Data from Zun Z, et al. *JACC: Cardiovasc Imag* 2011, 4:1253–1261)

**Fig. 8:**
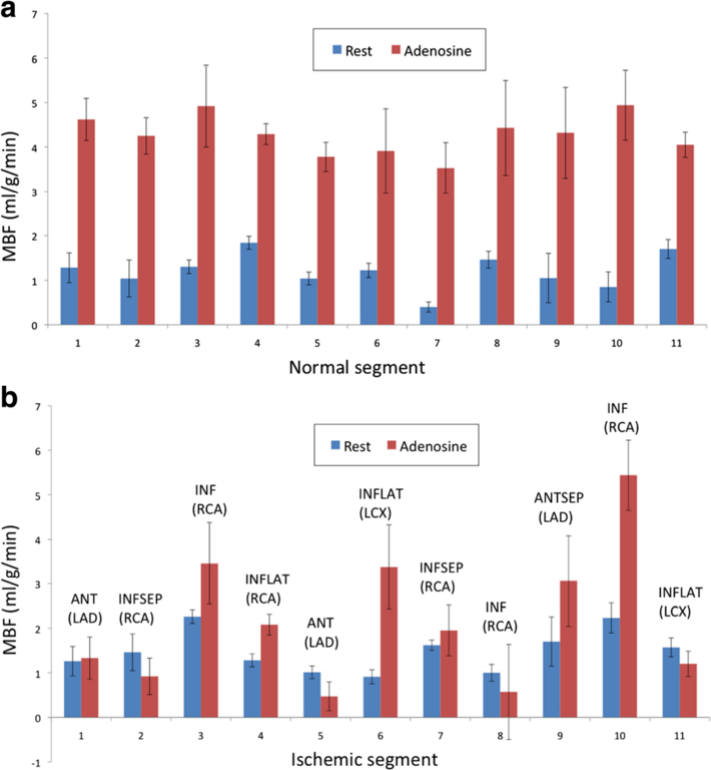
ASL-CMR pilot study in patients with suspected CAD demonstrated that ASL detected clinically relevant increases in MBF associated with vasodilation in (**a**) normal myocardium, and was able to differentiate those from (**b**) segments that were most ischemic based on coronary angiography. (Data from Zun Z, et al. *JACC: Cardiovasc Imag* 2011, 4:1253–1261)

To date, ASL-CMR in humans has not been validated against a gold standard such as PET. However, resting values have correlated well with PET literature, and the expected signal increases with stress have been observed. In particular, 30–40 % increases [84] were observed with isometric handgrip or leg elevation and 3–5x increases [85] were observed with vasodilation via intravenous adenosine. Zun et al. [86] performed ASL-CMR in both healthy human volunteers and patients with suspected CAD and reported clinically relevant changes in myocardial blood flow with vasodilation. Myocardial territories with low perfusion reserve (MBF_stress_/MBF_rest_) were also validated with coronary angiography. Two examples of CAD within the LAD and RCA found by ASL-CMR with their corresponding coronary angiograms are shown in Fig. 7. Perfusion reserve in normal and ischemic myocardial perfusion territories in 11 patients are shown in Fig. 8.

Northrup et al. [76] repeated ASL-CMR scans at 1.5 T and 3 T, and showed that although 3 T provided higher SNR (signal-to-thermal-noise ratio), it did not provide higher temporal SNR (signal-to-physiological-noise ratio). This interesting study suggests that unresolved sources of physiological noise limit sensitivity of ASL-CMR; an idea that was confirmed by a recent finding that shortening the imaging window via parallel imaging provides improved temporal SNR despite lower SNR [87]. Physiological noise remains as one of the important factors limiting sensitivity of the technique. Even small amounts of cardiac or respiratory motion can significantly corrupt the ASL difference image because inflowing blood signal is significantly smaller than background myocardial tissue signal. Most studies have tried to reduce physiological noise from cardiac and respiratory motion through breath-held, rapid single shot imaging during a stable cardiac phase. Physiological noise is measured as the variance of MBF from individual control and labeled image pairs. This calculation is straightforward using the signal intensity difference method at a single TI. Zun et al. [48] reported that physiological noise was 0.23 ml/g/min in 15 healthy volunteers. Physiological noise using the dual T1 approach is not directly measured because MBF from individual image pairs are never determined. Instead a surrogate for physiological noise is calculated from residuals of T1 curve fitting.

### Open questions

#### Absolute quantification and error sources

Quantification is a strength of ASL-CMR compared to first-pass techniques. The models as well as the post-processing algorithms are comparatively simple, since only water molecules are involved in the process, and since the labeling pulses create a perfectly reproducible bolus of well-known shape that is placed close to the imaged region. However, some uncertainties remain. Among them, the arterial transit time (ATT) from the labeled region to the entry of the capillary system may vary as a function of physiology. ATT becomes a significant source of error when it is much longer than the labeling delay time. Multi-delay protocols mitigate this source of error and can derive both ATT and MBF [20, 74]. The labeling efficiency affects the absolute MBF calculation in a direct proportional way. While variations of the labeling efficiency are likely negligible for the FAIR technique, the spASL technique is more prone to such errors. Other error sources are potential variations in T1 of tissue and blood if they are not measured along with perfusion. T1 variations in blood may be caused by differences in hematocrit.

### Complete coverage during stress

Current large animal and human ASL-CMR methods can capture a single slice in roughly 3 min of scan time. This has made it difficult to perform a comprehensive evaluation of ischemic disease which would require covering all coronary territories during the typical duration of peak vasodilation during a pharmacologic stress test (roughly 3 min for adenosine, less for regadenosin). The use of accelerated acquisition schemes including simultaneous multislice imaging [88, 89] or undersampled 3D acquisition [90–92] may be able to address this current limitation.

### High field imaging

Due to the increasing T1 with field strength, the life time of the magnetic labeling increases as well, and ASL is therefore a technique doubly benefiting from higher field in theory. This advantage is, however, compromised and yet unclear when considering the important contribution of physiological noise identified earlier [48, 76]. Whether high or even ultra-high field strengths (≥7 Tesla) can be used in a beneficial way for ASL-CMR will strongly depend on the ability of reducing motion contributions to signal variations observed in the myocardium.

### Motion correction

Beyond the most widely used ECG-triggered acquisition under breath-hold, available motion management techniques include post-processing image registration (rigid or non-rigid), but also advanced real-time techniques like motion tracking using a navigator. In ASL-CMR, motion tracking may be applied not only to the imaging slice, but it might also be useful to specific labeling volumes such as 2D spatial RF inversions [93]. Finally, advanced acquisition-reconstruction strategies like the Generalized Reconstruction by Inversion of Coupled Systems (GRICS) framework [94] directly integrate motion information collected during the acquisition into the reconstruction model and may be a way towards more efficient ASL-CMR acquisitions in the future.

### Clinical applications

**Fig. 9:**
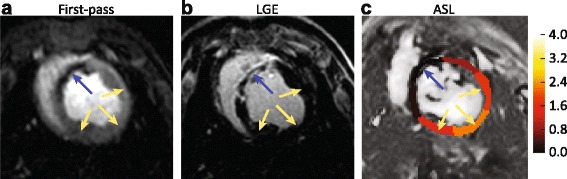
ASL-CMR in a pig with 4-week old septal infarct. **a** first-pass CMR image from peak myocardial enhancement. **b** Late Gadolinium Enhanced image and **c** ASL MBF map at rest. The color scale ranges from 0-4 ml/g/min. The infarcted myocardium (blue arrows) is thinned, and shows low MBF. Normal myocardium (yellow arrows) shows MBF of approximately 1.5 ml/g/min. (Data courtesy of Nilesh Ghugre, Sunnybrook Research Institute, University of Toronto, Toronto, Canada)

Due to its unlimited repeatability, ASL-CMR is an excellent candidate for perfusion stress testing. The technique can be used to assess and quantify regional myocardial perfusion and perfusion reserve in CKD or ESRD patients who suffer from higher rates of cardiovascular events, but cannot tolerate contrast agents required by other perfusion imaging techniques. However, challenges in applying ASL methods in the clinical setting remain. Current ASL methods require precise timing of labeling and imaging to the cardiac cycle, which is difficult to perform in the setting of cardiac arrhythmias. Developments in self gated, ECG free CINE imaging can be applied to ASL to help overcome this limitation [95, 96]. In disease processes with extremely low coronary flow such as in heart failure, severe coronary stenosis, or high coronary collateralization, ATT may be much longer that the labeling delay resulting in marked loss of the ASL signal. Velocity selective arterial spin labeling, which is insensitive to ATT by labeling blood based on its velocity within the arterioles adjacent to tissue, have been used successfully in the brain to and may be adapted for cardiac ASL [97, 98]. While not a direct measurement for viability and scar, ASL can potentially detect regions of hypo-perfusion associated with scar and can become a surrogate for scar localization with improvements in the spatial resolution of the technique. Figure 9 shows that rest ASL-CMR is comparable with rest first-pass CMR at detecting scar in a porcine model of myocardial infarction [99]. Finally, ASL-CMR may be used to assess diffuse disease processes such as coronary microvascular disease and diffuse interstitial fibrosis that result from diabetes, hypertension, and cardiomyopathy because it interrogates tissue perfusion directly.

### Conclusion

ASL-CMR is a safe technique for mapping regional myocardial perfusion and perfusion reserve. It does not require any contrast agents, is compatible with stress testing, and can be performed repeatedly and even continuously, with no incremental risk to the subject. ASL-CMR has been performed with great success in small-animals, and is the current method of choice when overall scan time is not a constraint. Its application to large animals and humans has been limited primarily by physiological noise (per-segment measurement variation approximately ±0.2 ml/g/min). It remains an active area of research, with many possible solutions on the horizon. Successful developments in brain ASL and small-animal ASL-CMR have helped to inform many of the most recent developments in large animal and human ASL-CMR.

## Abbreviations

1.5 T: 1.5 Tesla
2D: 2-dimensional
3 T: 3 Tesla
ASL: arterial spin labeling
ATT: arterial transit time
BBB: blood - brain barrier
BLOSM: block low-rank sparsity with motion guidance
CAD: coronary artery disease
CBF: cerebral blood flow
CHD: coronary heart disease
CKD: chronic kidney disease
CMR: cardiovascular magnetic resonance
ECG: electrocardiogram
ESRD: end-stage renal disease
EPI: echo-planar Imaging
EPISTAR: EPI signal tagging with alternating radiofrequency
FAIR: flow-sensitive alternating inversion recovery
FLASH: fast low angle shot
GBCA: Gadolinium-based contrast agents
Gd: Gadolinium
GRAPPA: generalized autocalibrating partially parallel acquisitions
GRE: gradient echo
HR: heart rate
k-t PCA: k-space-time principle component analysis
k-t SENSE: k-space-time sensitivity encoding
LLFAIR: Look-Locker flow-sensitive alternating inversion recovery
LLFAIRGE: Look-Locker flow-sensitive alternating inversion recovery respiratory gated
LV: left ventricle
MBF: myocardial blood flow
MBV: myocardial blood volume
nNOS: neuronal nitric oxide synthase
PASL: pulsed arterial spin labeling
PCASL: psuedo-continuous arterial spin labeling
PET: positron emission tomography
PN: physiological noise
RF: radiofrequency
SNR: signal - to - noise ratio
spASL: steady pulsed arterial spin labeling
SPECT: single-photon emission computed tomography
SSFP: steady - state free precession
Time-SLIP: time-spatial inversion pulse
